# Integrative analysis of gut microbiota composition, host colonic gene expression and intraluminal metabolites in aging C57BL/6J mice

**DOI:** 10.18632/aging.101439

**Published:** 2018-05-16

**Authors:** Benthe van der Lugt, Fenni Rusli, Carolien Lute, Andreas Lamprakis, Ethel Salazar, Mark V. Boekschoten, Guido J. Hooiveld, Michael Müller, Jacques Vervoort, Sander Kersten, Clara Belzer, Dieuwertje E.G. Kok, Wilma T. Steegenga

**Affiliations:** 1 Division of Human Nutrition, Wageningen University and Research, 6708 WE, Wageningen, The Netherlands; 2 Nutrigenomics and Systems Nutrition, Norwich Medical School, University of East Anglia, Norwich NR4 7UA, United Kingdom; 3 Laboratory of Biochemistry, Wageningen University and Research, 6708 WE, Wageningen, The Netherlands; 4 Laboratory of Microbiology, Wageningen University and Research, 6708 WE, Wageningen, The Netherlands

**Keywords:** aging, gut microbiota, metabolites, colonic gene expression, host-microbe interactions

## Abstract

The aging process is associated with diminished colonic health. In this study, we applied an integrative approach to reveal potential interactions between determinants of colonic health in aging C57BL/6J mice. Analysis of gut microbiota composition revealed an enrichment of various potential pathobionts, including *Desulfovibrio* spp*.*, and a decline of the health-promoting *Akkermansia* spp*.* and *Lactobacillus* spp. during aging. Intraluminal concentrations of various metabolites varied between ages and we found evidence for an increased gut permeability at higher age. Colonic gene expression analysis suggested that during the early phase of aging (between 6 and 12 months), expression of genes involved in epithelial-to-mesenchymal transition and (re)organization of the extracellular matrix were increased. Differential expression of these genes was strongly correlated with *Bifidobacterium* spp. During the later phase of aging (between 12 and 28 months), gene expression profiles pointed towards a diminished antimicrobial defense and were correlated with an uncultured *Gastranaerophilales* spp. This study demonstrates that aging is associated with pronounced changes in gut microbiota composition and colonic gene expression. Furthermore, the strong correlations between specific bacterial genera and host gene expression may imply that orchestrated interactions take place in the vicinity of the colonic wall and potentially mediate colonic health during aging.

## Introduction

Aging is a complex process characterized by a time-dependent loss of physical fitness accompanied by an increased risk of morbidities [1]. The increase in life expectancy and the increased prevalence of age-related pathologies [1] demands further insight into the mechanisms underlying the aging process.

The colon is mainly involved in absorption of water and nutrients, but also plays an important role in general body health [2]. Together with the small intestine, the colon constitutes the largest part of the whole-body immune system and is critical for an appropriate immune response towards the continuous exposure to potential inflammatory stimuli [3]. One of the unique features of the colon is that it harbors a complex ecosystem of micro-organisms referred to as the gut microbiota. The gut microbiota is crucial for the development and maturation of the intestinal immune system during the early phase of life, as well as the maintenance of the intestinal epithelial barrier [4]. Furthermore, the gut microbiota is involved in fermentation of non-digestible fibers and the production of short-chain fatty acids (SCFAs) [5], and as such lives in symbiosis with the host. In a healthy situation, the host is able to arrange an appropriate immune response upon microbial stimuli, which is non-responsive towards beneficial commensal bacteria, but reacts to pathogenic bacteria and restricts bacterial overgrowth [6,7]. However, aging is accompanied by a decline in function of the immune system and a chronic low-grade inflammation, known as immunosenescence and inflammaging, respectively [8]. These factors, together with age-related changes in diet, lifestyle as well as colon physiology, inevitably trigger changes in gut microbiota composition [9]. Alterations in gut microbiota composition have been linked to intestinal inflammatory diseases, as well as the development of other pathologies, e.g. metabolic syndrome, frailty and nervous system diseases [10–12]. Thus, in the context of mechanisms underlying healthy aging, gaining knowledge on novel molecular interactions between gut microbiota and the host may be the key for the prevention and treatment of age-related pathologies.

Remarkable age-related changes in gut microbiota composition were observed in large human cohort studies [10,13–15]. However, interpretation of the data is complex because of a wide range of interfering lifestyle factors, including diet and medication, but also other factors, such as genetic background and place of residence. Furthermore, the microbiota composition in these studies was analyzed in faecal samples, while previous studies showed distinct bacterial populations in faeces and colon lumen [16–20]. This observation raises the question whether the microbiota composition in fresh faeces is representative for the microbiota composition in the colon. Besides, when exploring the effects of the microbiota composition on colonic gene expression profiles, the analysis of the colonic luminal content is preferred, because of its close proximity to the colonic wall. However, a major advantage of using faecal samples is the ability to investigate changes in gut microbiota composition during aging in a longitudinal manner.

To deepen our understanding regarding the effects of aging while limiting the effects of interfering factors, we generated an aging cohort of male C57BL/6J mice. These mice received a semi-synthetic diet, were individually housed and fresh faeces was collected from the same mice at 4, 8, 12, 18, 24 and 28 months for analysis of faecal microbiota composition. After sacrifice at 6, 12, 24 and 28 months of age, scrapings of the colonic wall were isolated to investigate colonic gene expression and colonic luminal content was collected for investigation of colonic microbiota composition and metabolomics analysis.

We hypothesized that the interaction between host and the gut microbiota will change during the aging process and might contribute to a decline in colonic health at old age. To obtain potential mechanistic insights that could explain transcriptional perturbations in response to changes in colonic microbiota composition during aging, or vice versa, we used a comprehensive integrative analysis to explore interactions between the gut microbiota and host colonic gene expression.

## RESULTS

### Physiological differences between mice during aging

Body weight as well as food intake increased during aging (Figure S1A-B). Also liver weight and relative liver weight (% of body weight) were significantly higher in older mice (Figure S1C-D). To determine if metabolic health altered during aging, fasting insulin levels in plasma were assessed. These levels were significantly increased at 24 and 28 months compared to 6 months (p<0.01) (Figure S1E). Death rates of the mice remained low till the age of 20 months and started to increase afterwards (Figure S1F). Most mice that died prematurely suffered from multiple conditions (Data set S1).

### Alterations in faecal and colonic microbiota composition during aging

**Figure 1:**
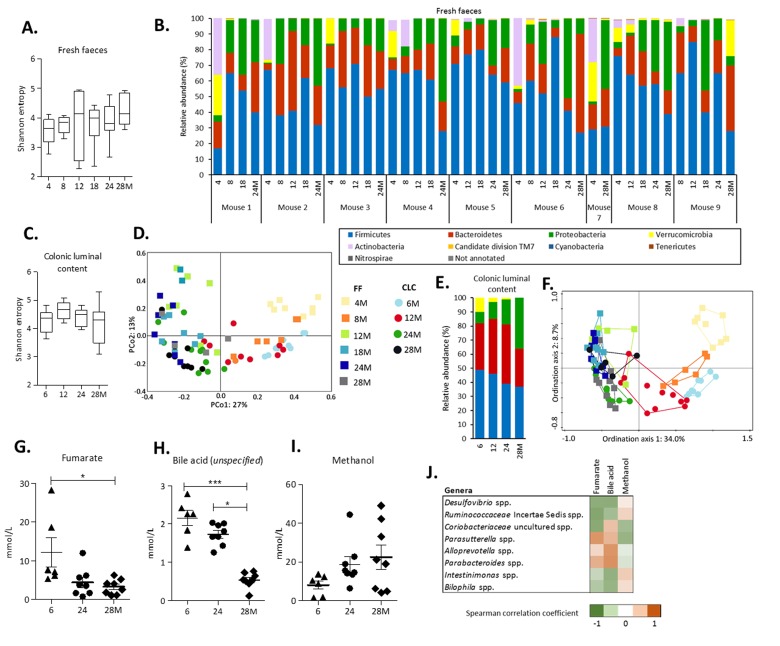
**Alterations in gut microbiota composition and metabolites during aging.** (**A**) The alpha-diversity (Shannon entropy) measured by the Shannon entropy in fresh faeces. Boxes extend from the 25th to 75th percentile, line in the middle represents median, and whiskers represent minimum and maximum values. (**B**) Relative abundance (%) at phylum level in fresh faeces collected longitudinally in a subset of 9 mice. (**C**) The alpha-diversity (Shannon entropy) measured by the Shannon entropy in colonic luminal content. **(D)** Principal Coordinate Analysis (PCoA) based on Bray-Curtis distances showing dissimilarities between all individual samples (FF and CLC). (**E**) The average relative abundance (%) at phylum level in colonic luminal content. Legend corresponds to graph with relative abundance at phylum level in fresh faeces. (**F**) Redundancy Analysis (RDA) displaying the part of the variation in microbiota composition explained by the age groups and source. Sample plot showing the clustering of the samples. Legend corresponds to PCoA plot. The distance between the ages and sample sources approximates the average dissimilarity of the microbiota composition. The FF analyses were based on a subset of n=9 mice at consecutive ages (4, 8, 12, 18, 24 and 28 months). For the CLC analyses, n=10 mice per age group (6, 12, 24 and 28 months) were included. (**G**) Levels of fumarate, **(H)** an unspecified bile acid and **(I)** methanol detected by 1H-NMR in CLC at 6 months (n=6), 24 months (n=8) and 28 months (n=8) of age. Error bars represent standard error of the mean (S.E.M**.**)**.** (**J**) Strongest Spearman correlation coefficients (-0.6<r>0.6) for the concentrations of the identified metabolites and the relative abundances of the 50 most abundant genera in colonic luminal content.

Fresh faeces samples were collected longitudinally at 4, 8, 12, 18, 24 and 28 months of age in a subset of 9 mice. Sequencing of the V3-V4 region of the 16S rRNA gene revealed that the alpha-diversity, in terms of Shannon entropy, did not change during aging (Figure 1A). To explore variance in faecal microbiota composition, a Principal Component Analysis (PCA) on relative abundance of genera was performed. The PCA revealed that the first principal component, explaining 42.7% of the variation, separated the mice aged 4 and 8 months from the other age groups (Figure S2A). Dissimilarities in faecal microbiota composition between age groups were further investigated at two taxonomic levels. At the phylum level, the relative abundance of *Proteobacteria* was less abundant in young mice (4 months) and increased from 8 months onwards in most mice (Figure 1B). *Verrucomicrobia* and *Actinobacteria* were highly abundant at 4 months, but both disappeared in most mice between 4 and 12 months of life (<1%). By exception, the relative abundance of *Verrucomicrobia* in mouse 9 at 28 months of age was high compared to the other mice at the same age (Figure 1C).

At genus level, the higher relative abundance of the *Proteobacteria* phylum in older mice was largely accounted for by a significantly higher abundance of the pathobionts *Desulfovibrio* spp*.* and *Bilophila* spp*.* (Table 1). However, the strongest aging-related change within this phylum was the significantly decrease in relative abundance of *Parasutterella* spp*.* during aging. The lower relative abundance of the *Verrucomicrobia* phylum was solely explained by *Akkermansia* spp*.* that significantly decreased during aging (Table 1). The relative abundance of *Bifidobacterium* spp*.* and *Coriobacteriaceae* spp*.* both decreased strongly and significantly explaining the age-dependent decrease in relative abundance of the *Actinobacteria* phylum. Finally, although the phyla *Bacteroidetes* and *Firmicutes* in itself did not significantly change during aging, several genera belonging to these phyla showed strong and significant changes between young and old mice (Table 1 and Table S1).

**Table 1:** Relative abundance of genera (abundance threshold ≥0.1% in a least one sample) that strongly changed (-10>FC>10) in either fresh faeces (FF) and/or colonic luminal content (CLC) between young and old mice (FDR p-value<0.1)

		**Fresh Faeces**								**Colonic Luminal Content**					
**Phylum**	**Genus**	**4M**	**8M**	**12M**	**18M**	**24M**	**28M**	**Fold change^1^**	**FDR p-value^2^**	**6M**	**12M**	**24M**	**28M**	**Fold change^3^**	**FDR p-value^2^**
Firmicutes	*Anaerovorax* spp*.*	0.001	0.003	0.009	0.015	0.015	0.02	18.3	0.024	0.007	0.019	0.014	0.035	4.7	0.063
	*Bacillus* spp*.*	0	0	0	1	0.251	0.991	>1000.0	0.035	0	0.19	0.356	0.944	>1000.0	0.223
	*Clostridium sensu stricto 1* spp.	0.008	0.129	0.056	0.268	0.207	0.213	28.0	0.024	0.123	0.16	0.155	0.367	3.0	0.363
	*Coprococcus* spp.	0	0	0.001	0.001	0.004	0.002	181.0	0.081	0.005	0.002	0.006	0.024	4.3	0.814
	*Lactobacillus* spp.	13	0.481	0.868	1	0.249	0.34	-38.2	0.024	0.447	0.157	0.129	0.139	-3.2	0.109
	*Roseburia* spp.	0.019	0.089	0.347	0.424	0.607	0.868	46.9	0.024	0.107	0.209	0.386	0.547	5.1	<0.001
	*Turicibacter* spp.	0.071	2	5	16	7	4	56.7	0.024	0.163	1	2	2	12.3	0.007
	uncultured *Christensenellaceae* spp.	0.002	0.011	0.024	0.026	0.021	0.051	23.3	0.024	0.007	0.007	0.014	0.024	3.5	0.040
	uncultured *vadinBB60* spp*.*	0.045	1	2	0.8	0.771	1	22.4	0.024	3	10	8	6	2.0	1.000
	unidentified *vadinBB60* spp.	0	0.002	0	0	0	0.029	>1000.0	0.200	0.057	0.113	0.012	0.002	-32.1	0.007
Bacteroides	*Odoribacter* spp.	0.54	3	3	5	4	7	13.0	0.024	6	14	14	6	1.0	0.884
	*RC9 gut group* spp.	0.682	4	2	2	2	9	13.2	0.024	3	2	6	5	1.7	0.144
Proteobacteria	*Bilophila* spp.	0.004	0.049	0.031	0.078	0.065	0.056	12.9	0.024	0.02	0.03	0.049	0.075	3.8	0.006
	*Desulfovibrio* spp.	2	14	10	25	33	25	12.5	0.024	8	12	18	36	4.5	<0.001
	*Parasutterella* spp.	0.196	0.072	0.074	0.005	0	0.004	-54.1	0.024	0.246	0.185	0.002	0.003	-93.2	<0.001
Verrucomicrobia	*Akkermansia* spp.	12	0.768	0.84	0.181	0.128	6	-2.0	0.024	10	3	1	0.114	-87.7	<0.001
Actinobacteria	*Bifidobacterium* spp.	19	4	1	0.051	0.07	0.027	-701.1	0.044	0.651	0.112	0.011	0.077	-8.5	0.003
	uncultured *Coriobacteriaceae* spp.	1	0.077	0.148	0.046	0.036	0.015	-69.0	0.024	0.078	0.014	0	0.008	-9.9	<0.001
Candidate division TM7	*Candidatus Saccharimonas* spp.	0.005	0.012	0.111	0.087	0.035	0.067	12.3	0.024	0.013	0.064	0.024	0.039	3.0	0.907
Cyanobacteria	uncultured *Gastranaerophilales* spp.	0	0	0	0.001	0.001	0	0.0	0.200	0.048	0.069	0.008	0.004	-10.9	0.044

Number of mice: Fresh faeces 4M: n=8; 8M: n=6; 12M: n=7; 18M: n=8; 24M: n=8; 28M: n=5. Colonic luminal content 6M: n=10; 12M: n=10; 24M: n=10; 28M: n=10.

^1^Fold change (FC) of relative abundance in fresh faeces samples between young (4 months) and old (28 months) old mice. ^2^ Determined by a Wilcoxon Signed Rank Test or Kruskal-Wallis Test (performed in SPSS) for fresh faeces and colonic luminal content, respectively. The adjusted p-values are shown, corrected for multiple testing using the Benjamini-Hochberg method. A False Discovery Rate of 0.1 was considered as significant. ^3^Fold change (FC) of relative abundance in colonic luminal content samples between young (6 months) and old (28 months) old mice.

Next to the analysis of the fresh faeces samples, the microbiota composition of the colonic luminal content of 40 mice sacrificed at the age of 6, 12, 24 and 28 months of age was investigated. Similar to the results obtained from the fresh faeces samples, no significant changes in Shannon entropy were found (Figure 1C) and the PCA revealed that the youngest and oldest age groups explained most of the variation (Figure S2B). These findings were strengthened by the distance-based Principal Coordinate Analysis (PCoA), since the first principal coordinate, explaining 27% of the variation, also separated young and old mice (Figure 1D). Comparison of the relative abundance at phylum and genus level between fresh faeces and colonic luminal content revealed mostly similar trends, although *Actinobacteria* phylum was hardly abundant in colonic luminal content (Figure 1E, Table 1, Figure S3). To investigate the impact of both age and source (fresh faeces and colonic luminal content) on the total variation, a Redundancy analysis (RDA) was performed. Ordination axis 1 explained 34.0% of the variation and separated the youngest age groups of both sources. Interestingly, ordination axis 2 explained 8.7% of the variation and separated the colonic luminal content and fresh faeces samples, indicating that the explanatory variable age contributed more to the variation in microbiota composition than the source variable (Figure 1F). Taken together, these results reveal pronounced changes in gut microbiota composition during aging, both in fresh faeces and more moderate in colonic luminal content. Additionally, subtle differences between the faecal and colonic microbiota composition were found.

### Metabolite profiles in colonic luminal content

To explore whether observed changes in gut microbiota composition may come with potential functional consequences, ^1^H-NMR was performed on colonic luminal content at 6, 24 and 28 months of age. Concentrations of the SCFAs butyrate, propionate and succinate did not differ between the age groups, except for acetate that was significantly lower at 24 months compared to 6 months (p<0.05) (data not shown). Intraluminal concentrations of fumarate decreased during aging, as well as an unspecified bile acid (Figure 1G-H). It was not possible to exactly identify this bile acid, since this peak in the NMR spectrum (0.17 ppm) is located in a rather unexplored region. However, it can be ruled out that this is one of the bile acids as described by Kok and colleagues [21], since these bile acids were identified in a different region of the spectrum (0.6-0.8 ppm). The concentration of methanol increased in older mice (Figure 1I). A correlation analysis was performed to investigate whether the levels of these metabolites could be related to the relative abundances of the 50 most abundant genera. The strongest negative correlations were found for fumarate that was negatively correlated with the relative abundance of *Desulfovibrio* spp. (r=-0.651), an uncultured *Ruminococcaceae* spp. (r=-0.708) and an uncultured *Coriobacteriaceae* spp. (r=-0.681), and positively correlated with *Parasutterella* spp. (r=0.637) (Figure 1J).

### Changes in colonic gene expression in 6, 12, 24 and 28-month-old mice

**Figure 2:**
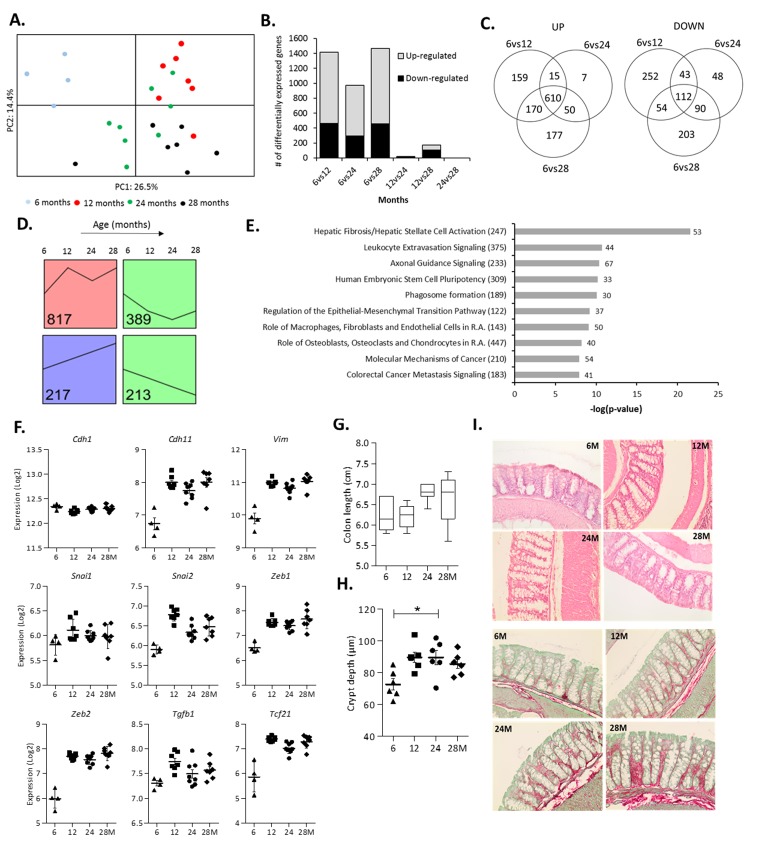
**Changes in colonic gene expression in 6, 12, 24 and 28-month-old mice.** (**A**) Principal Component Analysis (PCA) plot using the top 1,000 most variable genes showing the first 2 principal components. (**B**) The number of differentially expressed genes for all age comparisons (IBMT q<0.01). (**C**) Venn diagram showing the number of overlapping and unique differentially expressed genes between 6vs12, 6vs24 and 6vs28 months. (**D**) Each significantly differentially expressed gene (6vs12, 6vs24, 6vs28 months, q<0.01) was assigned to the model profile that most closely matched their gene expression profile, executed by STEM. The model profiles with the highest number of genes assigned to (as indicated by the number in the box) are shown. (**E**) Top 10 most significant canonical pathways between 6 and 12 months as determined by Ingenuity Pathway Analysis (R.A. = Rheumatoid Arthritis). Numbers behind bars represent the number of differentially expressed genes (IBMT q<0.01) and numbers behind the pathways represent the total number of genes. (**F**) The gene expression (Log2) of the epithelial marker E-cadherin (*Cdh1*), mesenchymal markers OB-cadherin (*Cdh11*) and Vimentin (*Vim*), transcription factors Snail Family Transcriptional Repressor 1 and 2 (*Snai1, 2*) and Zinc Finger E-Box Binding Homeobox 1 and 2 (*Zeb1, 2*), Transforming growth factor beta 1 (*Tgfb1*) and Transcription Factor 21 (*Tcf21*). (**G**) Length of the colon (cm) at sacrifice. (**H**) Depth of colonic crypts (μm) measured in H&E stained colon samples. **(I)** Representative pictures of Haematoxylin and Eosin (H&E) staining and Sirius Red/Fast Green staining of colon tissue at 6, 12, 24 and 28 months (200x magnification). Error bars reflect standard error of mean (S.E.M.). *p<0.05.

To explore the effect of aging on gene expression in the colonic wall, microarray analysis was performed on mRNA isolated from colonic scrapings from the same mice for which we also had data on the microbiota composition. A PCA carried out on the top 1,000 most variable genes revealed a clear segregation of the 6-months-old mice (Figure 2A). No clear separate clustering was observed for the mice from the other age groups, indicating that only gene expression at 6 months differed substantially from all other age groups. The highest number of significantly differentially expressed genes was found when comparing the youngest (6 months) versus the three older age groups (Figure 2B). There was a large overlap of genes (n=610) that had an increased expression when comparing 6 months with 12, 24 or 28 months (Figure 2C). Together, these results indicate that the expression levels of a substantial number of genes increased between 6 and 12 months, and remained increased also at higher ages.

### Dominant cluster of genes similarly expressed between 6 and 12 months

Visualization of the expression profiles of the 1,990 genes that were differentially expressed in the colonic wall of 6-months-old mice and the three older age groups (Data set S2) revealed a clustering of the majority of genes in four different expression profiles (Figure 2D). The profile in red contained by far the largest number of genes (817 genes) and showed the most prominent expression change between 6 and 12 months, remaining mostly stable afterwards (Figure 2D). The green profiles (602 genes in total) and the purple profile (217 genes) represent genes with decreased and increased expression levels during aging, respectively. Gene Ontology (GO) enrichment analysis of the green and purple profiles did not reveal any significant GO categories. However, analysis of the red profile revealed numerous significant GO categories that were all related to the extracellular matrix (ECM) organization and other structural development processes (Table 2). Ingenuity Pathway Analysis (IPA) and Gene Set Enrichment Analysis (GSEA) carried out on the genes differentially expressed between 6 and 12 months confirmed that similar pathways were affected (Figure 2E and Table S2A). IPA also revealed the ‘Regulation of the Epithelial-to-Mesenchymal Transition Pathway’ as a significantly regulated pathway in the colon between 6 and 12 months of age (Figure 2E and Table S2C). To analyze this aspect in more detail we assessed the expression regulation of genes involved in the epithelial-to-mesenchymal transition (EMT) in our microarray data set (Table S2D). Remarkably, while the expression of the epithelial markers E-cadherin (*Cdh1*), Occludin (*Ocln*) and most Claudins was minimally altered, the expression levels of the mesenchymal markers OB-cadherin (*Cdh11)*, Vimentin (*Vim*), Fibronectin (*Fn1*) and Vitronectin (*Vtn*) (Figure 2F and Table S2D) were strongly increased between 6 and 12 months and remained constant afterwards. In addition, the same expression pattern was found for transcription factors associated with EMT, i.e. Snail Family Transcriptional Repressor 1 and 2 (*Snai1, 2*), Zinc Finger E-Box Binding Homeobox 1 and 2 (*Zeb1, 2*) (Figure 2F), as well as other EMT-inducers, such as Transforming growth factor beta 1 (*Tgfb1*) and Transcription Factor 21 (*Tcf21*). IPA identified the transcription factor Transforming growth factor beta 1 (Tgfb1) as the strongest activated upstream regulator associated with differential gene expression between 6 and 12 months (Table S2E).

**Table 2:** Top 5 most significant Gene Ontology (GO) categories (i.e. Biological Process, Molecular Function, Cellular Component) belonging to the red, purple and combined green profiles, as determined by STEM.

Category ID		Category Name	#Genes Category		#Genes Assigned					#Genes Expected			#Genes Enriched		p-value			Corrected p-value*	
RED PROFILE																			
GO:0031012		extracellular matrix	157		125					64.5			60.5		9.70E-25			4.30E-21	
GO:0072359		circulatory system development	229		162					94			68		4.30E-22			1.90E-18	
GO:0005578		proteinaceous extracellular matrix	129		103					53			50		1.50E-20			6.80E-17	
GO:0072358		cardiovascular system development	175		129					71.8			57.2		4.80E-20			2.20E-16	
GO:0001944		vasculature development	175		129					71.8			57.2		4.80E-20			2.20E-16	

PURPLE PROFILE																		
GO:0007416	synapse assembly			28		11		3.1			7.9			8.10E-05		0.363		
GO:0022607	cellular component assembly			307		52		33.5			18.5			3.10E-04		1		
GO:0044085	cellular component biogenesis			317		52		34.6			17.4			7.20E-04		1		
GO:0050808	synapse organization			45		12		4.9			7.1			2.30E-03		1		
GO:0031514	motile cilium			10		5		1.1			3.9			2.30E-03		1		
							
COMBINED GREEN PROFILES																		
GO:0005654		nucleoplasm		197			87		59.6			27.4		9.50E-06			0.043	
GO:0031981		nuclear lumen		253			105		76.5			28.5		3.10E-05			0.139	
GO:0070013		intracellular organelle lumen		279			114		84.4			29.6		3.10E-05			0.139	
GO:0043233		organelle lumen		279			114		84.4			29.6		3.10E-05			0.139	
GO:0044428		nuclear part		286			114		86.5			27.5		1.20E-04			0.517	

*Bonferroni correction

### Change in colon morphology during aging

To study the potential morphological consequences of the observed changes in ECM- and EMT-related genes we measured the colon length at sacrifice as well as colonic crypt depths in H&E-stained Swiss rolls. The obtained results revealed an increase in colon length during aging (Figure 2G). A marked deepening of the crypts between 6 and 12 months of age was observed, which minimally increased afterwards (Figure 2H). Furthermore, Sirius Red/Fast Green staining of the same Swiss rolls showed that the ECM protein collagen gradually increased with age (Figure 2I).

### Integrative analysis of microbiota composition and gene expression in the early phase of aging

**Figure 3:**
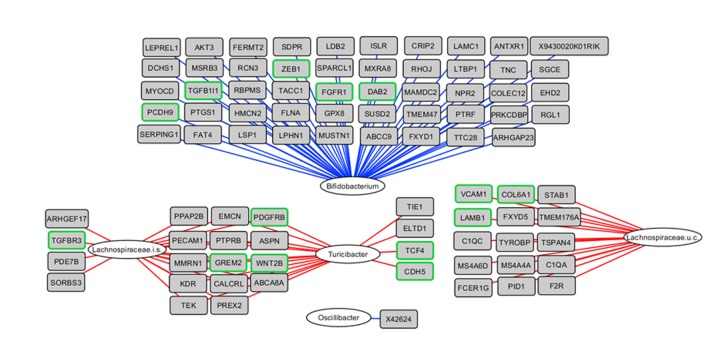
**Integrative analysis of colonic microbiota composition and gene expression in the early phase of aging.** The 817 up-regulated genes as determined by STEM were correlated with the 50 genera with a relative abundance ≥0.1% in at least 1 sample, including the mice aged 6 and 12 months. Relevance network showing the genera (white circles) and genes (grey rectangles) that were correlated (r<-0.80 or r>0.80). Red lines represent positive correlations, blue lines represent negative correlations. Regarding the negative correlations, only the top 50 strongest negative correlations are shown for visualization purposes. Genes with green frames are involved in EMT and/or ECM-related processes. i.s.=incertae sedis; u.c.: uncultured.

To obtain insight into the correlations between differential gene expression during the early phase of aging and the colonic microbiota composition, an integrative analysis was performed based on data of the individual mice. The 50 genera with a relative abundance of more than 0.1% in at least one sample and the 817 genes identified by STEM (Figure 2D, red cluster) showed several strong (r<-0.80 or r>0.80) positive and negative correlations (top 25 strongest correlations shown in Figure 3). *Bifidobacterium* spp*.* was negatively correlated with 228 genes, including a cluster of EMT- and ECM-associated genes, e.g. *Cdh11*, *Fn1*, *Zeb1*, *Zeb2*, *Vim,* *Vtn* and *Fgfr1*, amongst others (Figure 3 and Data set S3). Additionally, *Turicibacter* spp*.* and an uncultured member of the *Lachnospiraceae* family were positively correlated with a large number of genes, including Platelet Derived Growth Factor Receptor Beta (*Pdgfrb*), Wnt Family Member 2B (*Wnt2b*) and Gremlin 2 (*Grem2*), amongst others. Together, these results might imply the potential role of gut microbiota in the regulation of ECM (re)organization and EMT, or vice versa.

### Aging-specific changes in colonic gene expression between 12 and 28 months

**Figure 4:**
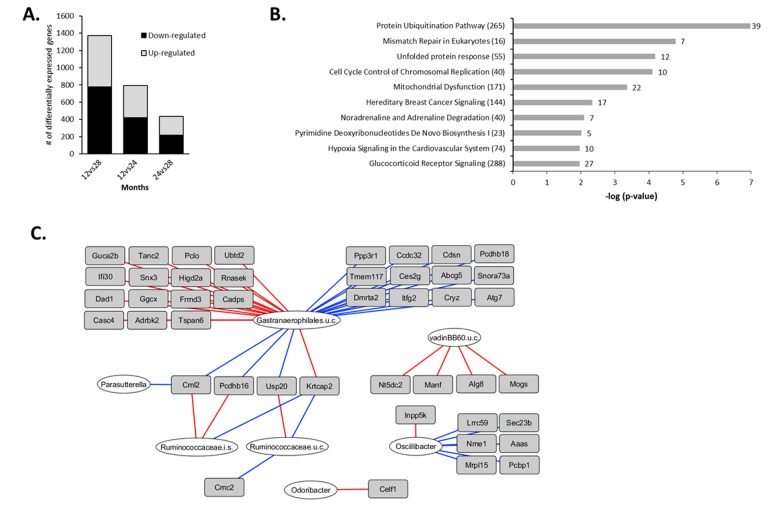
**Aging-specific changes in colonic gene expression between 12 and 28 months and integration with colonic microbiota composition.** (**A**) Number of differentially expressed genes between 12vs28, 12vs24 and 24vs28 months (IBMT p<0.01). (**B**) Top 10 most significant canonical pathways between 12 and 28 months identified by Ingenuity Pathway Analysis (IPA). Numbers behind bars represent the number of differentially expressed genes (IBMT p<0.01) and numbers behind the pathways represent the total number of genes. (**C**) Correlations between colonic microbiota composition and gene expression during aging. The 1371 genes differentially expressed between 12 and 28 months were correlated with the 50 genera with a relative abundance ≥0.1% in at least 1 sample, including the mice aged 12 and 28 months. Relevance network showing the genera (white circles) and genes (grey rectangles) that were correlated (r<-0.80 or r>0.80). Red lines represent positive correlations, blue lines represent negative correlations. For visualization purposes, only the top 25 strongest positive top 25 negative correlations are shown. i.s.=incertae sedis; u.c.: uncultured.

Next, we investigated alterations in gene expression during the later phase of the aging process by analyzing the changes between 12 and 28 months of age. Since the number of differentially expressed genes (n=170, q<0.01) was too low to perform a functional analysis, a less stringent IBMT p-value<0.01 was applied to include genes displaying more subtle expression changes, resulting in 1,371 significantly differentially expressed genes (Figure 4A). The top 15 highest up- and down-regulated genes presented in Table 3 show that Fatty acid binding protein 6 (*Fabp6*) had the strongest increased expression. The top 15 down-regulated genes were dominated by immune- and inflammatory-related genes, including 8 immunoglobulin-coding genes, Angiogenin 4 (*Ang4*), regenerating islet-derived 3 beta (*Reg3b*) and Resistin like beta (*Retnlb*). Another interesting immune-related gene displaying age-related reduced expression, but with a smaller fold change ranking them out of the top-15 list, was Indoleamine 2,3-dioxygenase 1 (*Ido1*) (Data set S4).

**Table 3:** Top 15 highest up-regulated and down-regulated genes between 12 and 28 months.

	**Top 15 up-regulated genes**	**FC**	**p-value**	**q-value**
*Fabp6*	fatty acid binding protein 6	3.29	1.59E-05	2.25E-03
*Cml2*	N-acetyltransferase 8 (GCN5-related) family member 2	2.33	4.42E-09	1.25E-05
*1700057G04Rik*	RIKEN cDNA 1700057G04 gene	2.28	2.54E-04	1.30E-02
*Creb3l3*	cAMP responsive element binding protein 3-like 3	2.14	2.62E-06	7.64E-04
*Cml5*	N-acetyltransferase 8 (GCN5-related) family member 5	1.99	1.02E-04	7.95E-03
*Cyp2c67*	cytochrome P450, family 2, subfamily c, polypeptide 67	1.89	3.83E-03	5.02E-02
*Pcdhb18*	protocadherin beta 18	1.88	5.11E-11	5.80E-07
*Snora44*	small nucleolar RNA, H/ACA box 44	1.88	1.05E-04	8.03E-03
*Pcdhb16*	protocadherin beta 16	1.86	1.38E-08	3.14E-05
*Abcg5*	ATP binding cassette subfamily G member 5	1.80	9.10E-06	1.69E-03
*Gm6086*	galactose-3-O-sulfotransferase 2C	1.79	5.69E-04	1.92E-02
*Abcc2*	ATP binding cassette subfamily C member 2	1.77	3.61E-04	1.53E-02
*Snora73b*	small nucleolar RNA, H/ACA box 73B	1.73	5.47E-06	1.29E-03
*Npc1l1*	NPC1 like intracellular cholesterol transporter 1	1.70	2.51E-03	4.11E-02
*Abcg8*	ATP binding cassette subfamily G member 8	1.70	1.90E-05	2.49E-03
				
	**Top 15 up-regulated genes**	**FC**	**p-value**	**q-value**
*Igkv8-24*	immunoglobulin kappa chain variable 8-24	-7.63	9.26E-04	2.48E-02
*Ang4*	angiogenin, ribonuclease A family, member 4	-4.79	1.93E-05	2.49E-03
*Ighg3*	immunoglobulin heavy constant gamma 3	-3.63	4.46E-04	1.68E-02
*Iglv2*	immunoglobulin lambda variable 2	-3.50	1.73E-03	3.53E-02
*Reg3b*	regenerating islet-derived 3 beta	-3.30	9.94E-03	8.25E-02
*Iglv1*	immunoglobulin lambda variable 1	-3.26	2.01E-04	1.17E-02
*Igkv3-12*	immunoglobulin kappa variable 3-12	-3.21	9.55E-03	8.04E-02
*Iglc2*	immunoglobulin lambda constant 2	-2.94	9.64E-04	2.54E-02
*Igkv4-57*	immunoglobulin kappa variable 4-57	-2.61	8.44E-04	2.32E-02
*Retnlb*	resistin like beta	-2.59	9.42E-05	7.64E-03
*Dhrs9*	dehydrogenase/reductase (SDR family) member 9	-2.17	2.22E-04	1.22E-02
*Cadps*	Ca2+-dependent secretion activator	-1.98	6.26E-08	7.37E-05
*Iglv3*	immunoglobulin lambda variable 3	-1.92	6.12E-04	1.97E-02
*Frmd3*	FERM domain containing 3	-1.83	1.36E-07	1.41E-04
*2310079G19Rik*	RIKEN cDNA 2310079G19 gene	-1.82	3.99E-04	1.59E-02

The most significantly enriched down-regulated gene sets identified by GSEA were related to DNA replication and DNA synthesis (Table S3B). In addition, IPA identified ‘Protein Ubiquitination Pathway’, ‘Mismatch Repair in Eukaryotes’ and ‘Cell Cycle Control of Chromosomal Replication’ as most significantly regulated canonical pathways during the later phase of the aging process (Figure 4B) and X-box binding protein 1 (Xbp1) as the strongest inhibited upstream regulator (Table S3C). Taken together, these results indicate that during the later phase of aging (between 12 and 28 months), gene expression profiles pointed towards a diminished antimicrobial defense, an altered protein degradation response and aberrations in DNA repair mechanisms.

### Integrative analysis of microbiota composition and gene expression in the later phase of aging

A second integrative analysis was carried out to investigate the correlation between gene expression and colonic microbiota composition during the late phase of life. For that purpose, the data of the 50 genera with a relative abundance of more than 0.1% in at least one sample and the 1,371 genes that were differentially expressed between 12 and 28 months (IBMT p<0.01) for each individual mouse were integrated. The highest number of most pronounced correlations (r<-0.80 or r>0.80) was found for an uncultured member of *Gastranaerophilales* spp. (Figure 4C). *Pcdhb16, Pcdhb18* and *Abcg5,* all in the top 15 of strongest up-regulated genes (Table 3), as well as Ubiquitin Specific Peptidase 20 (*Usp20*), were negatively correlated with the relative abundance of this uncultured member. Interestingly, strong positive correlations (R≥0.75) were found for the uncultured member of *Gastranaerophilales* spp. with the down-regulated immune-related genes *Ang4*, *Retnlb* and *Ido1* (Data set S5).

### Markers of gut permeability increased during aging

**Figure 5:**
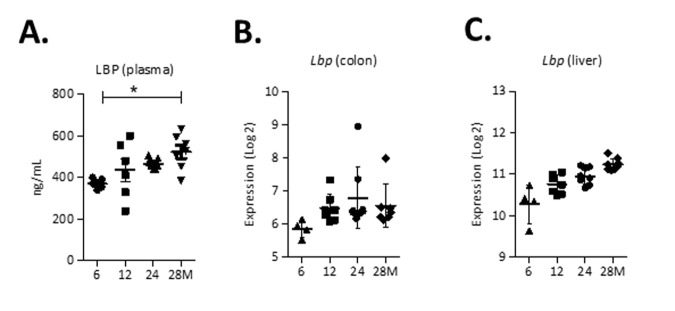
**Markers of gut permeability during aging.** (**A**) Concentrations of Lipopolysaccharide-binding protein (LBP) (ng/ml) in plasma. (**B**) Expression (Log2) of *Lbp* in the colon and (**C**) liver. *p<0.05.

To determine whether there were changes in markers of gut permeability, concentrations of lipopolysaccharide-binding protein (LBP), a marker for bacterial endotoxins, were assessed in plasma of mice aged 6, 12, 24 and 28 months. LBP concentrations were significantly higher (p<0.05) in plasma from mice aged 28 months compared to 6-months-old mice (Figure 5A). Besides, the microarray data of the colonic wall revealed increased expression levels of the *Lbp* gene during aging (Figure 5B). LBP is also synthesized as an acute-phase protein in the liver and therefore we assessed expression levels of *Lbp* in liver tissue, which were increased during aging (Figure 5C). Interestingly, although we found increased plasma markers of gut permeability at higher age, there was no collective increase in expression of tight junction genes (Data set S2).

## DISCUSSION

In the present study, we report that aging is associated with pronounced changes in gut microbiota composition and colonic gene expression. In agreement with the current literature, we found an increase in potential pathobionts and a decrease in health-promoting bacteria during aging, which could potentially contribute to the development of age-related pathologies [12,22]. In contrast to the mild effects of aging on colonic gene expression that we found several years ago [23], we now report much stronger effects, probably because we included older mice (28 months instead of 21 months of age). Moreover, by applying an integrative approach, we show that several bacterial genera strongly correlated with colonic gene expression, providing evidence for potential host-microbe interactions.

A unique feature of this study resides in the collection of fresh faeces at consecutive time points, allowing the observation of changes in microbiota composition during the life course in the same mouse. Because environmental circumstances were strictly controlled, a broad range of external factors could be disregarded, allowing us to focus primarily on aging-related changes. However, a limitation of the C57BL/6J mouse model in aging research is the increased vulnerability to premature hearing loss, impaired glucose tolerance and tumor formation, amongst others [24]. Besides, it should be taken into account that differences exist in gut microbiota composition between mice and humans, mainly because of differences in size of the intestinal tract, metabolic rate and diet [25].

We found a strong increased relative abundance of *Desulfovibrio* spp*.* and *Bilophila* spp., two closely related genera that produce the potentially toxic compound hydrogen sulfide [26]. It was previously shown that the relative abundance of *Desulfovibrio* spp. was increased in elderly humans compared to younger subjects [14]. Furthermore, increased abundance of both *Bilophila* spp. and *Desulfovibrio* spp. has been associated with mucosal inflammation [26,27], highlighting the presumable negative physiological consequences of an enrichment of these genera in the aged population. The increased relative abundance of these members of the gram-negative *Proteobacteria* could explain the significantly elevated plasma LBP levels at 28 months. LBP is an acute-phase protein that binds to LPS, resulting in an innate immune response [28]. We suggest that the shift in gut microbiota composition during aging eventually caused a diminished intestinal barrier function that favored the translocation of LPS into the systemic circulation. We also found a higher relative abundance of *Odoribacter* spp*.* and *Turicibacter* spp*.* at higher age. These genera may be involved in colon tumorigenesis, since they were previously found to be enriched in tumor-bearing mice [29]. Additionally, *Odoribacter* spp*.* was recently found to have a higher relative abundance in old compared to young mice [30]. *Turicibacter* spp. was less abundant in humans with Alzheimer Disease compared to non-demented participants [31].

We found that the relative abundance of *Akkermansia* spp*.*, *Bifidobacterium* spp*.* and *Lactobacillus* spp*.* decreased at middle-age, i.e. 8 months. Each of these genera has one or more health-promoting properties, such as the production of SCFAs, enforcement of the mucus layer and stimulation of the immune system [32–34]. Previous studies reported a loss of these health-associated genera in aged mice and humans [35,36], but our findings implicate that in mice, these genera disappear already at middle-age. It is important to note that *Akkermansia* spp*.* was shown to be negatively correlated with adiposity [37]. Accordingly, the observed decrease in relative abundance of this genus could be related to an increase in bodyweight with age. *Coprococcus* spp*., Roseburia* spp. and *Christensenellaceae* spp*.*, all described as producers of the health-promoting butyrate [38,39], had increased relative abundances in old mice compared to young mice in our study. This runs contrary to human studies in which these genera have shown lower relative abundance [14,40] and that elderly persons had a gut microbiota composition reflecting a lower butyrate production capacity [41]. However, *Christensenellaceae* spp*.* as well as *Akkermansia* spp. were detected in faeces of centenarians, i.e. people over 100 years of age [13,14], implicating that these genera could be linked to an extreme life span. Although the oldest mice in our study could not be considered as representative for centenarians, their age was extremely high for mice [42], possibly explaining the presence of this genus. We found relatively low concentrations of intraluminal butyrate and other SCFAs, which is possibly the consequence of the low fiber semisynthetic diets that we used.

An uncultured *Coriobacteriaceae* spp*.* followed a strong decreasing pattern during aging. The *Coriobacteriaceae* family is a commensal community in the gut and some members play a role in several metabolic processes, such as bile acid metabolism [43]. We did not find a strong correlation between the uncultured *Coriobacteriaceae* spp*.* with the level of the unspecified bile acid, but strong negative correlations were found with both fumarate and methanol. The relative abundance of *Parasutterella* spp*.*, which decreased strongly during aging in our study, was also found to be significantly reduced in patients with colorectal cancer [44], but has not been reported in relation to aging yet. We found that this genus was positively correlated with fumarate and negatively correlated with methanol. Since both the uncultured *Coriobacteriaceae* spp*.* and *Parasutterella* spp*.* showed strong decreasing abundances during aging and were correlated with various metabolites, it would be worthwhile to further investigate the role of these genera and metabolites in the aging process. A recent study discovered that the bacterial metabolite colanic acid promoted longevity in the host *Caenorhabditis elegans* [45], emphasizing the importance of further studies to focus on the mediating role of bacterial metabolites during aging.

In this study, we collected both faecal and colonic samples, allowing us to investigate the similarity in microbiota composition of both sample sources. Although age predominantly contributed to the variation in gut microbiota composition, we did observe subtle dissimilarities in relative abundances between sample sources. The most notable difference was the higher relative abundance of the *Actinobacteria* phylum in fresh faeces compared to colonic luminal content of young mice. A possible explanation for these dissimilarities is the fact that, in contrast to the colonic luminal content, fresh faeces passed the complete colon and may have been affected by various factors known to influence microbiota composition, such as oxygen levels and pH-values [16]. The reliability of the use of stool samples for assessment of microbiota composition in human studies is currently under debate. However, the subtle differences detected between sample sources in the present study do not directly demand for a switch in using the relatively non-invasive stool samples to colonic content isolates in aging studies.

Remarkably, both the highly abundant *Akkermansia* spp*.* and *Desulfovibrio* spp*.* displayed a strong age-related decrease or increase, respectively, but did not show strong correlations with colonic gene expression. On the other hand, *Bifidobacterium* spp*.* was negatively correlated with a considerable number of genes related to ECM and EMT processes. Strong positive correlations were also found for *Turicibacter* spp*.* and two uncultured *Lachnospiraceae* spp*.* with the same subgroup of genes. Interestingly, the expression of mesenchymal markers and related transcription factors was strongly increased between 6 and 12 months, while expression of epithelial markers was not or moderately affected. These findings suggest that a change in cellular composition of the tissue occurs, in terms of a gain of a mesenchymal phenotype, but without loss of the epithelial phenotype. Conceivably, the EMT was not of classical nature and the step of de-epithelialization was eliminated, a process called partial or intermediate EMT [46]. IPA identified *Tgfb1* as main upstream regulator which is a known and potent inducer of EMT during normal development, tissue repair, organ fibrosis and metastasis [47,48]. However, it is not plausible to assume that the observed EMT was pathological, since the mice remained in a in healthy condition far beyond the time point at which the EMT was induced, and death rates remained extremely low till the age of 20 months. More likely, the EMT contributed to normal development of the mice that was possibly induced by certain gut microbiota, since we hypothesize that the presence of *Bifidobacteria* spp*.* (or its metabolites, e.g. lactic acid) could weaken the expression of ECM- and EMT-related genes, or vice versa. It was previously shown that pathogens do have the ability to activate EMT-signaling pathways [49], and our results could indicate that also non-pathogens might be involved in the regulation of these pathways. Next, we hypothesize that the gain in mesenchymal markers resulted in an increased deposition of ECM [48,50], as confirmed by the collagen staining. Ultimately, these events could have led to a change in colon morphology in our study, as observed by the increase in colonic crypt depth.

By exploring the changes between 12 and 28 months, we found evidence for an altered protein degradation response, together with a potential aberration of DNA repair mechanisms in the colon during the late phase of life. These processes are common events during aging and are linked to several age-related pathologies [1]. Moreover, we observed a decreased intestinal immune response during aging, since several immunoglobulins, as well as the immunosuppressive *Ido1* gene, were strongly down-regulated. Besides, the strong down-regulation of *Ang4*, *Reg3b,* *Retnlb* and *Ido1* pointed toward a dysregulation of antimicrobial peptide expression at old age. Interestingly, *Ang4*, *Retnlb* and *Ido1* were positively correlated with an uncultured *Gastranaerophilales* spp. To the best of our knowledge, little is known about *Gastranaerophilales* spp*.*, however, genome sequencing revealed that this order is capable of converting glucose, mannose, starch, or glycogen into lactate, ethanol and formate [51]. Notably, the semi-synthetic diet fed to the mice in our study was rich in starch, possibly explaining the presence of this bacterial strain. *Usp20* was negatively correlated to the uncultured *Gastranaerophilales* spp*.*, highlighting the possible role of this bacterial strain in the observed altered protein degradation response during aging.

Taken together, this study demonstrates that aging is associated with pronounced changes in gut microbiota composition and colonic gene expression, without interference of environmental factors. A presumably deleterious shift in gut microbiota composition occurred during aging, as we found an increase in potential pathobionts and a decrease in health-promoting bacteria. However, we also reported strong changes in relatively unexplored genera, which might have health effects during the aging process. Next to the pronounced differences in gut microbiota and colonic gene expression during aging, we also found strong correlations between these two aspects of colonic health. This finding implies that host-microbe interactions might play an important role during aging and therefore the newly identified molecular interactions should be investigated more extensively in future research.

## MATERIALS AND METHODS

### Ethics statement

The institutional and national guidelines for the care and use of animals were followed and the Local Committee for Care and Use of Laboratory Animals at Wageningen University approved the experiment (code number: drs-2010151b).

### Mice and study design

In this study, 9-week old male C57BL/6J mice were housed individually and fed an ad libitum semi-synthetic (AIN-93W) diet. The study design was described previously in more detail [52]. In order to study the aging process, mice were randomly distributed into four groups that were sacrificed at 6, 12, 24 and 28 months (n=11-16 mice per group). For 4 mice in the 24-months group and 5 mice in the 28-months group, fresh faeces were consecutively collected (directly after defecation) at the age of 4, 8, 12, 18, 24 and 28 months. Bodyweight and food intake were monitored bi-weekly. During sacrifice, the colon was opened and the colonic luminal content was collected first. Then, the middle part of the colon (~1 cm) was turned inside out, rolled and embedded in paraffin (‘Swiss rolls’) for histology. From the remaining colon parts, the mucosa and submucosa were scraped for RNA isolation. The mice included in the present study were also used as control group for diet-intervention studies in the liver and colon of which the data have been reported in previous publications [21,52–54].

### Gut microbiota composition analysis

For both fresh faeces (42 samples) and colonic luminal content (40 samples) the microbiota composition was determined. The detailed methods have been described previously [21]. Briefly, DNA was isolated from faecal and colonic luminal content samples using the ZR Fecal DNA MicroPrep kit (ZYMO Research, Irvine, CA, USA) according to the manufacturer’s instructions. Lysis of the samples was performed by bead beating using ZR BashingBead™ Lysis Tubes (ZYMO Research, Irvine, CA, USA). Next, the V3-V4 region of the 16S rRNA gene was amplified. After purification of the amplicon, DNA quality was checked and a second PCR was performed using sample-specific barcoded primers (Nextera XT index kit, Illumina, San Diego, CA, USA). Purified PCR products were sent to BaseClear service laboratory (BaseClear BV, Leiden, The Netherlands) for sequencing on the MiSeq platform (Illumina, San Diego, CA, USA). The Casava pipeline (version 1.8.3, Illumina, San Diego, CA, USA) was used for de-multiplexing of the FASTQ files. After initial quality control, three fresh faeces samples were excluded for further analysis, as the coverage was below threshold (50% of the median number of reads). CLC Microbial Genomics Module version 1.2.1 (CLC Bio, Qiagen, Aarhus, Denmark) was used for further analysis of the sequencing data. FASTQ files of the fresh faeces and colonic luminal content were imported and processed simultaneously. The identified OTUs were aligned against the Silva database (version 119) at 97% similarity [55]. A number of 1,034,303 reads were detected, for which 1208 OTUs were identified. 25 of these OTUs (265 reads) were unavailable. Shannon entropy was considered as a metric for alpha-diversity and the beta-diversity was determined using Bray-Curtis distances. Principal Component Analyses and Redundancy analysis were carried out using Canoco 5 [56]. The input consisted of the 303 OTUs with a relative abundance of ≥0.1% in at least one sample. With regard to the RDA, the numbers of reads were centered and standardized using the Hellinger transformation [57]. The 16S rRNA gene data described in this study have been deposited in the sequence read archive at the NCBI with accession number SRP145060.

### Determination of metabolites in colonic luminal content

The colonic luminal content of mice aged 6 months (n=6), 24 months (n=8) and 28 months (n=8) was prepared as described previously [21] and levels of metabolites were determined using ^1^H-NMR. From the aligned spectra, integrals for SCFAs were identified. Besides, an untargeted approach was taken to identify metabolites that differed between age groups. After baseline correction, the concentrations of the metabolites were calculated by taking into account the dilution factor and number of hydrogen atoms. Correlations between concentrations of identified metabolites and the relative abundances of the 50 most abundant genera in colonic luminal content were determined using Spearman’s correlation.

### Gene expression analysis

For a number of 4 to 8 mice per age group, RNA was isolated from scrapings of the colonic wall and liver using TRIzol reagent (Invitrogen, Breda, The Netherlands). Isolated RNA was purified using RNeasy Micro columns (Qiagen, Venlo, The Netherlands) and total RNA yield (Nanodrop ND-1000, Nanodrop Products, Maarssen, The Netherlands) and RNA integrity (Agilent 2100 Bioanalyzer, Agilent Technologies, Amsterdam, The Netherlands) were measured. Only RNA was used that had a RNA integrity number (RIN) above 8.0 (Table S4). Purified RNA (100 ng per sample) was converted to cDNA and labelled using an Ambion WT expression kit (Life Technologies, Bleiswijk, The Netherlands). Microarray hybridization and analysis was performed as previously described [52]. Differences in colonic gene expression between the age groups were analyzed using the Intensity Based Moderated T statistics (IBMT), using q-values <0.01 as threshold for the comparison 6 versus 12 months and p-values <0.01 for the comparison 12 versus 28 months. Microarray data has been submitted to the Gene Expression Omnibus (GEO) at the NCBI, and is accessible under number GSE113257. The Principal Component Analysis plot was generated using MultiExperiment Viewer version 4.9.0. Ingenuity Pathway Analysis (IPA) was used for identification of canonical pathways and upstream regulators [58]. Gene Set Enrichment Analysis was performed to functionally interpret gene regulation [59]. A false discovery rate (FDR) q-value of <0.1 was considered significantly enriched. Short Time-series Expression Miner (STEM) version 1.3.11 [60] was used to identify gene expression profiles during aging, using the mean Robust Multichip Average (RMA) values as input.

### Histology

Swiss rolls of 6 mice per age group were cut in 5 micrometer sections and mounted on microscope slides (VWR Superfrost Plus Micro Slide). The sections were dewaxed multiple times with xylene, rehydrated in alcohol and stained with Haematoxylin/Eosin (H&E) or Sirius red/Fast green. Pictures of the stained sections were taken using a microscope with Olympus CellSense Entry software (Olympus Europe, Hamburg, Germany). For the H&E stained sections, the depths of the colonic crypts were measured using Olympus Cell^B software.

### Multivariate analyses for gut microbiota and gene expression

In order to integrate colonic microbiota composition and colonic gene expression, we performed multivariate analyses in R 3.3.1 using the MixOmics package [61]. Partial Least Squares (PLS) regression in the canonical mode was used to determine the bi-directional relationship between the two datasets. We used data from individual mice for whom both gene expression as well as the microbiota composition from colonic luminal content were available. For the MixOmics analysis early during aging (i.e. between 6 and 12 months of age), a number of 11 mice were included (6 months: n=4; 12 months: n=7). A number of 14 mice were included for the analysis using the genes differentially expressed between 12 and 28 months (p<0.01) (12 months: n=7; 28 months: n=7). The genera with a relative abundance of ≥0.1% in at least 1 sample were included, resulting in a number of 50 genera. First, the sequencing reads of the genera were transformed and zeros were imputed based on the centered log ratio (clr) using the aldex2 package with 1024 Monte Carlo permutations [62]. Gene expression data were included after transformation into log2 RMA intensities. Relevance networks with the strongest positive and negative correlations (R<-0.8 or R>0.8) were visualized using Cytoscape [63].

### Plasma measurements

Plasma levels of LBP were determined using the LBP ELISA Kit Mouse (kit OKBB00573, Aviva Systems Biology, San Diego, USA), following the manufacturer’s protocol. Plasma samples were diluted 100 times. Plasma levels of insulin were determined using the Mouse Adipokine kit (MADKMAG-71K, Merck Millipore, Darmstadt, Germany), according to the manufacturer’s protocol.

### Statistical analyses

Statistical analyses were, unless stated otherwise, carried out using GraphPad Prism 5.04 (GraphPad Software, San Diego, California, USA). The Kolmogorov-Smirnov test was used to test if data were normally distributed. In case of normal distribution, one-way analysis of variance (ANOVA) in combination with Tukey’s multiple comparisons test was used to test for differences between ages. In case the data were not normally distributed, the non-parametric Kruskal-Wallis one-way analysis of variance following a Dunn’s post hoc test and a Mann-Whitney test were used for independent samples. A Friedman test or Wilcoxon Signed Rank test were used in case samples were dependent. The Bonferroni or Benjamini-Hochberg approach were used to adjust for multiple testing, if appropriate. For all statistical tests, unless stated otherwise, a p-value of p<0.05 was considered as statistically significant.

## Supplementary Material

Supplementary Figures

Supplementary Tables

Data Set S1

Data Set S2

Data Set S3

Data Set S4

Data Set S5
