# Supplementary material for: Integrative analysis of gut microbiota composition, host colonic gene expression and intraluminal metabolites in aging C57BL/6J mice
Source: Aging (Albany NY). 2018 May 16;10(5):930–50. doi: 10.18632/aging.101439 (PMC5990381; doi:10.18632/aging.101439)
Supplement: Supplementary Figures [file aging-10-101439-s001.pdf]

SUPPLEMENTARY MATERIAL

Please browse the Full Text version to see:

Supplementary Tables S1-S4.

Data set S1. Table with causes of death of the mice that died prior to sacrifice.

Data set S2. Genes differentially expressed between 6vs12, 6vs24 and 6vs28 months (q<0.01).

Data set S3. All correlations between the 50 genera with a relative abundance ≥0.1% in at least 1 sample and the 817 up-regulated genes assigned to the red profile as determined by STEM. Orange cells contain r>0.8, blue cells contain r<-0.8.

Data set S4. All genes differentially expressed between 12 and 28 months of age (p<0.01).

Data set S5. All correlations between the of 50 genera with a relative abundance ≥0.1% in at least 1 sample and the 1371 genes differentially expressed between 12 and 28 months. Orange cells contain r>0.8, blue cells contain r<-0.8.

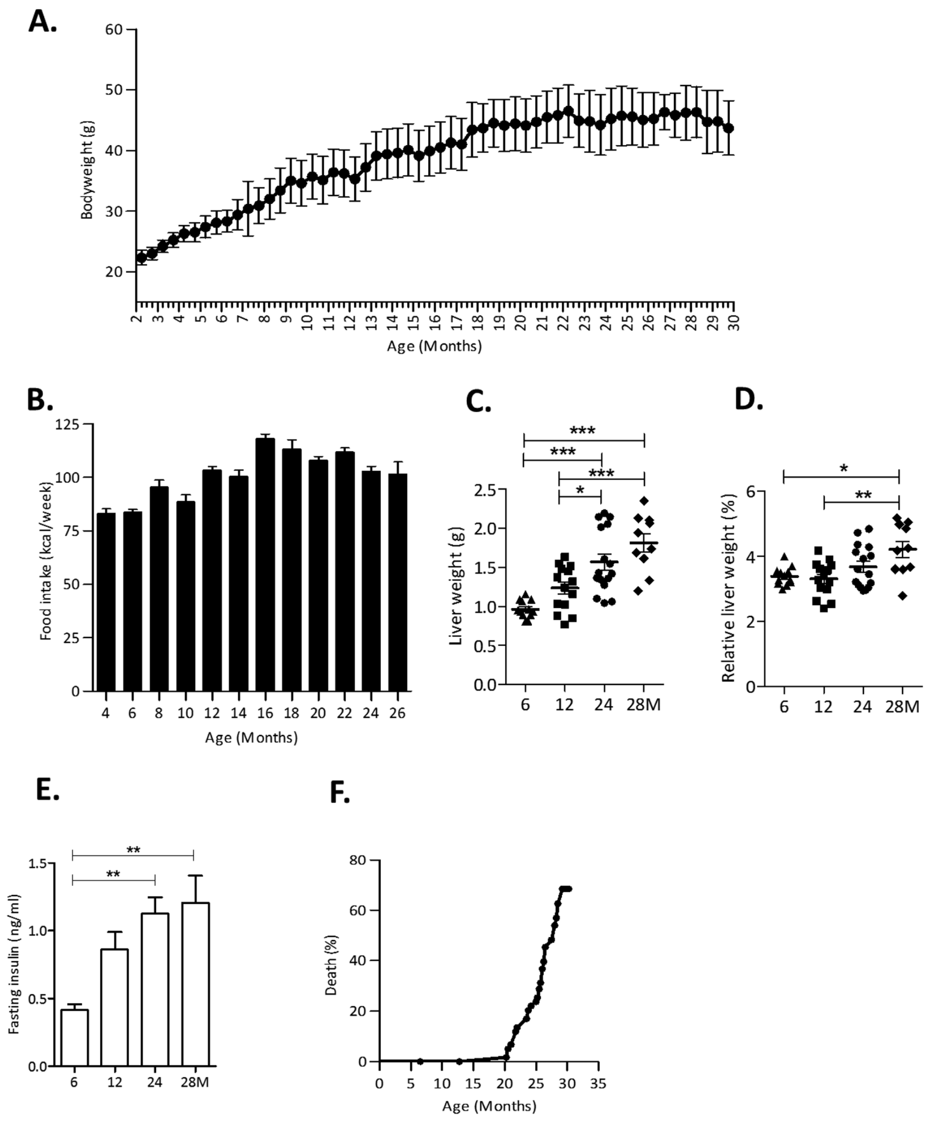

Figure S1. (**A**) Body weight measurements recorded every 2 weeks. (**B**) Mean food intake in kilocalories per week during the life span of the mice. (**C**) Liver weight in grams. (**D**) Relative liver weight as percentage of body weight. (**E**) Fasting insulin levels (ng/ml) measured in plasma. (**F**) Percentage of mice that died before sacrifice.

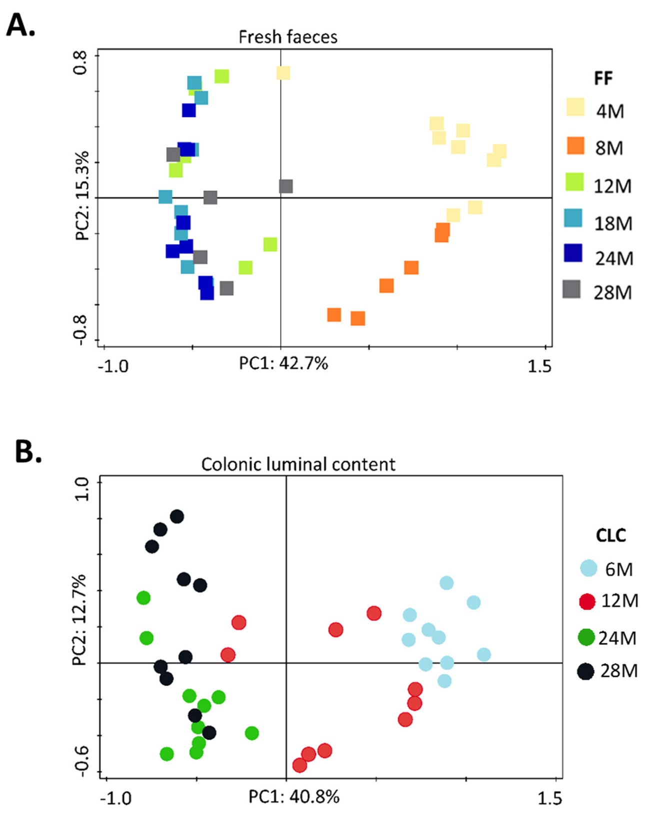

Figure S2. Figure (**A**) Principal Component Analysis (PCA) displaying separation of the fresh faeces (FF) samples collected at 4, 8, 12, 18, 24 and 28 months. (**B**) PCA displaying separation of the colonic luminal content (CLC) samples collected at sacrifice at 6, 12, 24 and 28 months.

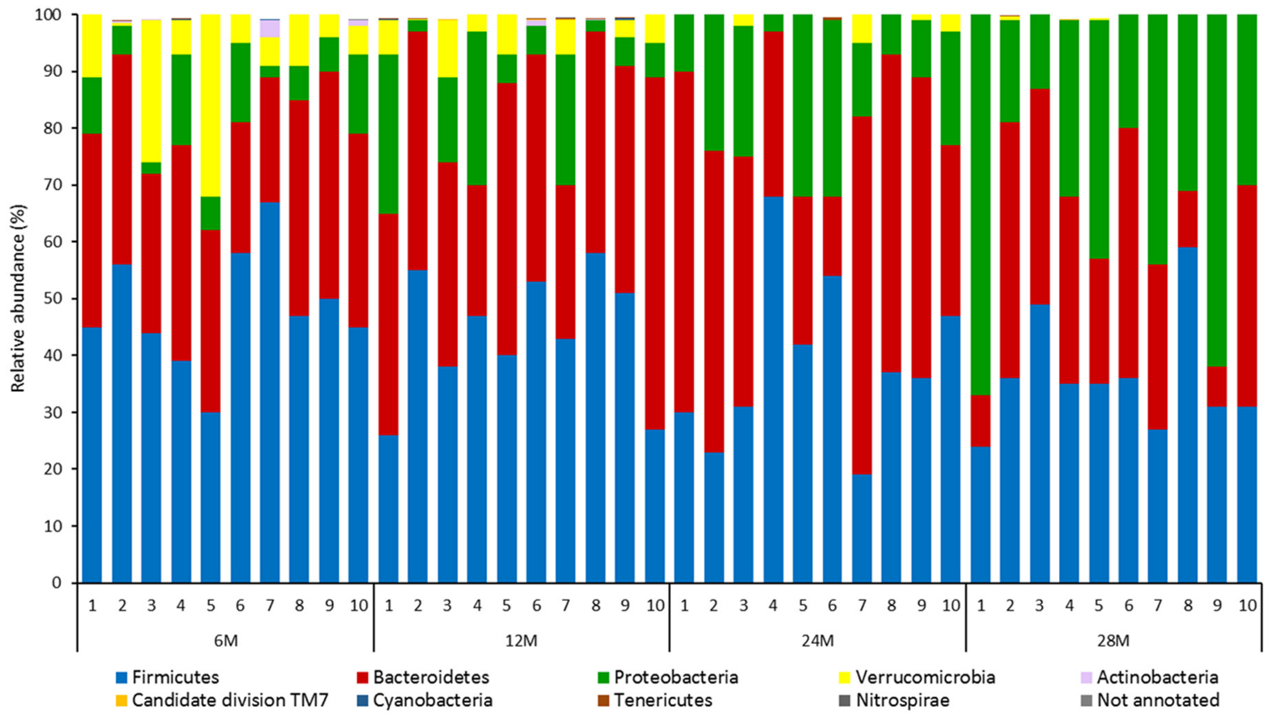

Figure S3. Relative abundance (%) at phylum level in colonic luminal content determined in all individual mice.

SUPPLEMENTARY REFERENCES

1. Kalluri R, Weinberg RA. The basics of epithelial-mesenchymal transition. J Clin Invest, 2009: 119: 1420-28.

2. Lamouille S, Xu J, Derynck R. Molecular mechanisms of epithelial-mesenchymal transition. Nat Rev Mol Cell Biol. 2014; 15:178-96.

3. Puisieux A, Brabletz T, Caramel J. Oncogenic roles of EMT-inducing transcription factors. Nat Cell Biol. 2014; 16: 488-94.

4. Serrano-Gomez SJ, Maziveyi M, Alahari SK. Regulation of epithelial-mesenchymal transition through epigenetic and post-translational modifications. Mol Cancer. 2016; 15:18.

5. Yu H, et al. The contribution of TGF-beta in Epithelial-Mesenchymal Transition (EMT): Down-regulation of E-cadherin via snail. Neoplasma. 2015; 62:1-15.

6. Zhao M, et al. dbEMT: an epithelial-mesenchymal transition associated gene resource. Sci Rep. 2015; 5: 11459.
