# Supplementary material for: Integrative analysis of gut microbiota composition, host colonic gene expression and intraluminal metabolites in aging C57BL/6J mice
Source: Aging (Albany NY). 2018 May 16;10(5):930–50. doi: 10.18632/aging.101439 (PMC5990381; doi:10.18632/aging.101439)
Supplement: Supplementary Tables [file aging-10-101439-s002.pdf]

SUPPLEMENTARY TABLES

Table S1. Relative abundance of the 50 genera (relative abundance threshold ≥0.1% in a least one sample) in fresh faeces and colonic luminal content. Genera indicated with an asterisk (*) had the strongest fold change between young and old mice and were selected for Table 1.

Phylum	Genus	FF-4M	FF-8M	FF-12M	FF-18M	FF-24M	FF-28M	FC FF 4vs28M[1]	FDR p-value[2]	CLC-6M	CLC-12M	CLC-24M	CLC-28M	FC CLC 6vs28M[3]	FDR p-value[2]
Firmicutes	*Allobaculum spp.*	28	46	44	26	29	17	-1.6	0.024	37	22	17	10	-3.7	0.000
	Anaerotruncus spp.	0.005	0.024	0.037	0.070	0.055	0.051	9.3	0.024	0.040	0.063	0.116	0.133	3.3	0.015
	*Anaerovorax spp.**	0.001	0.003	0.009	0.015	0.015	0.020	18.3	0.036	0.007	0.019	0.014	0.035	4.7	0.063
	*Bacillus spp.**	0	0	0	1	0.251	0.991	>1000	0.035	0	0.190	0.356	0.944	>1000	0.223
	Blautia spp.	12	5	7	2	3	3	-4.0	0.108	3	5	2	5	1.7	0.884
	*Clostridium sensu stricto 1 spp.**	0.008	0.129	0.056	0.268	0.207	0.213	28.0	0.024	0.123	0.160	0.155	0.367	3.0	0.363
	Coprococcus spp.*	0	0	0.001	0.001	0.004	0.002	181.0	0.081	0.005	0.002	0.006	0.024	4.3	0.814
	Enterococcus spp.	0.559	0	0	0	0	0	0	0.081	0	0	0	0	0	1.000
	Incertae Sedis *Defluviitaleaceae spp.*	0.052	0.082	0.173	0.369	0.283	0.215	4.1	0.024	0.034	0.095	0.105	0.238	7.0	0.003
	Incertae Sedis *Erysipelotrichaceae spp.*	0.036	0.012	0.004	0.021	0.010	0.056	1.6	0.074	0.026	0.007	0.011	0.018	-1.5	1.000
	Incertae Sedis family XIII *spp.*	0.011	0.011	0.013	0.032	0.038	0.071	6.5	0.024	0.017	0.011	0.022	0.026	1.5	0.907
	Incertae Sedis *Lachnospiraceae spp.*	0.029	0.080	0.087	0.170	0.186	0.213	7.3	0.024	0.106	0.199	0.246	0.432	4.1	0.003
	Incertae Sedis *Peptostreptococcaceae spp.*	0.075	0.534	0.408	0.555	0.412	0.448	6.0	0.024	0.691	0.692	0.687	0.354	-2.0	0.054
	Incertae Sedis *Ruminococcaceae spp.*	0.578	1	2	3	2	2	3.5	0.024	0.812	1	2	3	3.7	0.003
	Intestinimonas *spp.*	0.022	0.072	0.118	0.111	0.197	0.089	4.1	0.108	0.067	0.078	0.182	0.369	5.5	0.000
	*Lactobacillus spp.**	13	0.481	0.868	1	0.249	0.340	-38.2	0.024	0.447	0.157	0.129	0.139	-3.2	0.109
	Oscillibacter spp.	0.014	0.054	0.055	0.154	0.141	0.137	9.7	0.024	0.054	0.064	0.119	0.304	5.7	0.000
	Peptococcus spp.	0.004	0.045	0.033	0.131	0.064	0.092	21.2	0.159	0.030	0.056	0.042	0.058	1.9	0.724
	*Roseburia spp.**	0.019	0.089	0.347	0.424	0.607	0.868	46.9	0.024	0.107	0.209	0.386	0.547	5.1	0.000
	Ruminococcus spp.	0.009	0.007	0.021	0.002	0.008	0.062	7.1	0.200	0.012	0.017	0.048	0.059	5.1	0.003
	*Turicibacter spp.**	0.071	2	5	16	7	4	56.7	0.024	0.163	1	2	2	12.3	0.007
	uncultured *Christensenellaceae spp.**	0.002	0.011	0.024	0.026	0.021	0.051	23.3	0.024	0.007	0.007	0.014	0.024	3.5	0.040
	uncultured *Defluviitaleaceae spp.*	0.007	0.042	0.052	0.018	0.015	0.049	7.5	0.044	0.048	0.075	0.088	0.123	2.5	0.814
	uncultured *Erysipelotrichaceae spp.*	0.004	0	0	0.010	0	0	0	0.051	0.003	0.002	0.003	0.002	-1.5	0.400
	uncultured *Lachnospiraceae spp.*	0.249	0.693	2	2	2	2	8.0	0.024	0.788	2	2	2	2.5	0.006
	uncultured *Lachnospiraceae spp.*	0.037	0.033	0.006	0	0	0	0	0.051	0.055	0.078	0.004	0	0	0.000
	uncultured *Peptococcaceae spp.*	0.036	0.035	0.046	0.069	0.089	0.087	2.4	0.108	0.044	0.050	0.040	0.069	1.5	0.574

	uncultured *Peptostreptococcaceae spp.*	0.054	0.033	0.044	0.040	0.031	0.029	-1.9	0.024	0.114	0.483	0.356	0.359	3.1	0.678
	uncultured *Ruminococcaceae spp.*	0.687	2	2	5	4	3	4.4	0.024	2	2	3	5	2.5	0.007
	uncultured *vadinBB60 spp.**	0.045	1	2	0.800	0.771	1	22.4	0.024	3	10	8	6	2.0	1.000
	unidentified *vadinBB60 spp.**	0	0.002	0	0	0	0.029	>1000	0.200	0.057	0.113	0.012	0.002	-32.1	0.007
Bacteroidetes	*Alistipes spp.*	5	12	10	4	5	8	1.6	0.108	18	18	17	12	-1.5	0.044
	Alloprevotella spp.	0.138	0.040	0.074	0.110	0.089	0.051	-2.7	0.159	0.436	0.345	0.238	0.058	-7.5	0.000
	Bacteroides spp.	0.363	0.270	0.261	0.130	0.134	0.633	1.7	0.044	0.912	0.555	0.659	0.329	-2.8	0.011
	*Odoribacter spp.**	0.540	3	3	5	4	7	13.0	0.024	6	14	14	6	1.0	0.884
	Parabacteroides spp.	0.255	0.073	0.035	0.033	0.032	0.351	1.4	0.024	0.439	0.162	0.102	0.046	-9.6	0.000
	*RC9 gut group spp.**	0.682	4	2	2	2	9	13.2	0.024	3	2	6	5	1.7	0.144
	uncultured *Porphyromonadaceae spp.*	0.034	0.122	0.151	0.183	0.144	0.177	5.3	0.024	0.197	0.438	0.952	0.374	1.9	0.574
	uncultured *S24-7 spp.*	2	3	5	3	4	7	3.5	0.055	3	4	3	3	1.0	0.490
	uncultured *VC2.1 Bac22 spp.*	0.010	0.131	0.103	0.018	0.025	0.034	3.5	0.024	0.093	0.160	0.108	0.182	2.0	0.330
	unidentified *S24-7 spp.*	0	0	0	0	0	0	0	0.200	0.221	0	0	0	0	0.859
Proteobacteria	*Bilophila spp.**	0.004	0.049	0.031	0.078	0.065	0.056	12.9	0.024	0.020	0.030	0.049	0.075	3.8	0.006
	*Desulfovibrio spp.**	2	14	10	25	33	25	12.5	0.024	8	12	18	36	4.5	0.000
	*Parasutterella spp.**	0.196	0.072	0.074	0.005	0	0.004	-54.1	0.024	0.246	0.185	0.002	0.003	-93.2	0.000
Tenericutes	*Anaeroplasma spp.*	0	0	0	0	0	0	0	0.200	0	0	0.050	0	0	0.144
Verrucomicrobia	*Akkermansia spp.**	12	0.768	0.840	0.181	0.128	6	-2.0	0.024	10	3	1	0.114	-87.7	0.000
Actinobacteria	*Bifidobacterium spp.**	19	4	1	0.051	0.070	0.027	-701.1	0.044	0.651	0.112	0.011	0.077	-8.5	0.003
	uncultured *Coriobacteriaceae spp.**	1	0.077	0.148	0.046	0.036	0.015	-69.0	0.024	0.078	0.014	0	0.008	-9.9	0.000
Candidate division TM7	*Candidatus Saccharimonas**	0.005	0.012	0.111	0.087	0.035	0.067	12.3	0.024	0.013	0.064	0.024	0.039	3.0	0.907
Cyanobacteria	uncultured *Gastranaerophilales spp.**	0	0	0	0.001	0.001	0	0	0.200	0.048	0.069	0.008	0.004	-10.9	0.044

Number of mice: Fresh faeces 4M: n=8; 8M: n=6; 12M: n=7; 18M: n=8; 24M: n=8; 28M: n=5. Colonic luminal content 6M: n=10; 12M: n=10; 24M: n=10; 28M: n=10.
[1]Fold change (FC) of relative abundance in fresh faeces samples between young (4 months) and old (28 months) old mice. [2]A Wilcoxon Signed Rank Test and Kruskal-Wallis Test were performed in SPSS for fresh faeces and colonic luminal content, respectively. The adjusted p-values are shown, corrected for multiple testing using the Benjamini-Hochberg method. A False Discovery Rate of 0.1 was considered as significant. [3]Fold change (FC) of relative abundance in colonic luminal content samples between young (6 months) and old (28 months) old mice.

Table S2. (**A**) Table including the top 10 of significantly enriched up-regulated and (**B**) down-regulated gene sets between 6 and 12 months, as determined by GSEA. (**C**) Genes included in the canonical pathways 'Hepatic Fibrosis/Hepatic Stellate Cell Activation' and 'Regulation of the Epithelial-Mesenchymal Transition Pathway' identified by IPA. (**D**) Expression of genes described to be up- or down-regulated during EMT, selection based on literature (see references in supplemental data). (**E**) IPA identified the top 5 upstream regulators with the highest activation score between 6 and 12 months.

A.

NAME	SIZE	NES	FDR q-value
EXTRACELLULAR MATRIX ORGANIZATION	251	3.21	<0.001
COLLAGEN BIOSYNTHESIS AND MODIFYING ENZYMES	62	2.81	<0.001
ECM PROTEOGLYCANS	53	2.78	<0.001
ELASTIC FIBRE FORMATION	38	2.77	<0.001
KEGG ECM RECEPTOR INTERACTION	87	2.73	<0.001
COLLAGEN FORMATION	82	2.68	<0.001
INTEGRIN CELL SURFACE INTERACTIONS	64	2.66	<0.001
PLATELET DEGRANULATION	76	2.57	<0.001
KEGG FOCAL ADHESION	205	2.57	<0.001
MOLECULES ASSOCIATED WITH ELASTIC FIBRES	29	2.55	<0.001

B.

NAME	SIZE	NES	FDR q-value
OLFACTORY SIGNALING PATHWAY	308	-1.88	0.242
KEGG MATURITY ONSET DIABETES OF THE YOUNG	26	-1.85	0.170
TRAF6 MEDIATED IRF7 ACTIVATION	29	-1.81	0.196
RIG I MDA5 MEDIATED INDUCTION OF IFN ALPHA BETA PATHWAYS	67	-1.78	0.207
TRANSCRIPTIONAL ACTIVITY OF SMAD2 SMAD3 SMAD4 HETEROTRIMER	37	-1.72	0.324
KEGG TYPE II DIABETES MELLITUS	50	-1.71	0.289
DOWNREGULATION OF SMAD2 3 SMAD4 TRANSCRIPTIONAL ACTIVITY	19	-1.70	0.287
SMAD2 SMAD3 SMAD4 HETEROTRIMER REGULATES TRANSCRIPTION	25	-1.70	0.252
WP431 NUCLEAR RECEPTORS IN LIPID METABOLISM AND TOXICITY	30	-1.69	0.258
RORA ACTIVATES CIRCADIAN GENE EXPRESSION	24	-1.68	0.256

NES: Normalized Enrichment Score

C.

Hepatic Fibrosis / Hepatic Stellate Cell Activation		Regulation of the Epithelial-Mesenchymal Transition Pathway
MYH10	*FLT1*	*LOX*
CTGF	*EDNRB*	*TCF4*
MMP13	*COL6A2*	*SNAI2*
TGFBR2	*COL12A1*	*FGF2*
TGFB1	*FLT4*	*NCSTN*
MYL4	*COL1A1*	*PARD6G*
COL27A1	*CSF1*	*FZD1*
TIMP2	*EDNRA*	*KLB*
PDGFRB	*KDR*	*TGFBR2*
SMAD2		*FGF10*
COL4A1		*TGFB1*
FGFR1		*KL*
IL6R		*HGF*
MMP2		*TGFB2*
IGFBP5		*AKT3*
IFNAR2		*IRS2*
MYL9		*FZD2*
IGF2		*FGF7*
COL6A3		*PDGFRB*
ACTA2		*ETS1*
IGFBP3		*SMAD2*
TGFB3		*NOTCH3*
COL3A1		*FGFR1*
IGFBP4		*WNT2B*
FN1		*MMP2*
ICAM1		*ZEB1*
COL4A6		*PIK3R3*
PDGFA		*FZD4*
FGF2		*mir-192*
LEPR		*ZEB2*
COL4A2		*PIK3R6*
MYH11		*TGFB3*
COL15A1		*FZD5*
FAS		*TCF7L2*
COL5A1		*CLDN3*
COL1A2		*PSEN1*
COL6A1		*WNT5A*
IGF1		
HGF		
TGFB2		
PDGFRA		
COL18A1		
COL5A2		
VCAM1		

D.

TRANSCRIPTIONAL FACTORS		
Gene name	Fold Change	IBMT q-value
↑Ets2	1.03	3.57E-01
↑Junb	1.04	5.16E-01
↑Lef1	1.22	3.23E-02
↑Mkl1	-1.05	2.99E-01
↑Mkl2	-1.04	3.13E-01
↑Prrx1	1.04	3.92E-01
↑Snai1	1.23	7.72E-02
↑Snai2	**1.84**	2.09E-06
↑Twist1	1.14	1.63E-01
↑Twist2	1.18	9.86E-02
↑Zeb1	**2.04**	1.50E-05
↑Zeb2	**3.28**	2.07E-09
↑Tcf3	-1.02	5.27E-01
↑Tcf21	**2.89**	2.37E-07
↑Foxc2	1.05	4.55E-01

GROWTH FACTORS & OTHER SIGNALS		
Gene name	Fold Change	IBMT q-value
↑Ctgf	**2.07**	3.22E-04
↑Dab2	**2.33**	4.26E-07
↑Egflam	1.29	6.56E-03
↑Emr1	**2.32**	5.87E-08
↑Emr4	1.1	1.97E-01
↑Fgf1	-1.1	2.41E-01
↑Hgf	**2.16**	1.42E-03
↑Igf1	**2.6**	1.83E-07
↑Igfbp3	**3.68**	1.83E-08
↑Il6	-1.03	4.20E-01
↑Notch1	-1	6.15E-01
↑Notch2	1.12	2.41E-01
↑Notch3	1.45	1.03E-03
↑Notch4	1.11	1.87E-01
↑Pdgfa	-1.2	5.33E-03
↑Pdgfb	1.24	1.97E-02
↑Pdgfra	**2.92**	3.73E-08
↑Pdgfrb	**1.78**	1.31E-07
↑Shh	1.01	6.01E-01
↑Tgfb1	1.35	1.02E-02
↑Tgfb2	**1.81**	1.26E-04
↑Tgfb3	**1.53**	2.36E-03
↑Tgfbr1	1.11	6.15E-02
↑Tgfbr2	1.23	3.80E-04
↑Tgfbr3	**3.21**	8.78E-09
↑Vegfa	-1.06	3.11E-01
↑Wnt2	1.04	4.83E-01
↑Wnt2b	**2.89**	6.29E-09
↑Wnt5a	1.44	1.24E-02

REGULATION AT RNA LEVEL & HISTONE MODIFICATIONS		
Gene name	Fold Change	IBMT q-value
↑Dot1l	-1.01	5.69E-01
↓Esrp1	-1.07	8.70E-02
↓Esrp2	-1.01	5.86E-01
↑Kdm1a	-1.09	2.21E-02
↑Kdm3a	-1.24	2.87E-03
↑Mbnl1	1.07	1.01E-01
↓Mir124a-2	-1.13	1.90E-01
↓Mir124a-3	-1.06	3.14E-01
↑Mir155	-1.11	9.81E-02
↓Mir194-1	-1.2	6.06E-02
↓Mir1a-1	-1.02	4.79E-01
↓Mir1a-2	-1.04	3.11E-01
↓Mir200a	-1.37	2.22E-02
↓Mir200b	**-1.53**	4.12E-02
↓Mir200c	-1.14	2.45E-01
↓Mir205	-1.14	2.34E-01
↑Mir24-2	-1.11	2.21E-01
↓Mir29b-1	-1.11	6.62E-02
↓Mir30a	-1.35	2.94E-02
↓Mir34a	-1.11	3.20E-01
↓Mir34b	-1.05	4.54E-01
↓Mir34c	1.03	4.76E-01
↓Mir429	**-1.54**	1.26E-02
↓Mir491	1.12	2.73E-01
↑Mir9-1	-1.18	9.84E-02
↑Mir9-2	-1.21	3.25E-01
↑Mir9-3	-1.01	5.95E-01
↑Rbfox2	-1.01	5.92E-01
↓Rbm47	-1.09	2.55E-02
↑Srsf1	1.1	4.49E-02

↑ Up-regulated during EMT

↓ Down-regulated during EMT

(yellow) q<0.01

(orange) q<0.05

MARKER GENES					
Gene name	Fold Change	IBMT q-value	Gene name	Fold Change	IBMT q-value
↑Pik3ca	-1.08	7.84E-02	↑Bmp4	**3.21**	5.15E-09
↓Pkp2	-1.03	4.23E-01	↑Cav1	**2.92**	8.39E-06
↓Pkp3	-1.05	3.33E-01	↑Cd274	**-1.34**	2.63E-03
↓Pkp4	1.04	3.76E-01	↑Ddr2	**2.61**	3.74E-06
↑Pou5f1	-1.03	5.22E-01	↑Edn1	**-1.91**	1.16E-03
↑Rac1	-1	6.00E-01	↑Ednra	**2.75**	7.72E-08
↑Rho	1.07	3.40E-01	↑Lcn2	**2.44**	6.04E-03
↑Rhoa	1.01	5.84E-01	↑Postn	**4.16**	3.13E-10
↑Bmi1	1.06	3.85E-01	↑Smad2	-1.21	1.03E-03
↑Cdc42	1.03	3.27E-01	↑Smad3	-1.14	2.03E-02
↓Cdh1	-1.07	3.49E-02	↑Smad4	-1.07	3.47E-01
↑Cdh2	1.24	1.08E-01	↑Smad5	-1.02	4.88E-01
↓Cldn1	**1.55**	1.28E-02	↑Sox2	-1.03	5.01E-01
↓Cldn25	1.1	1.37E-01	↑Sparc	**3**	9.76E-08
↑Axl	**3.07**	1.03E-08	↑Vcan	**2.33**	4.65E-06
↓Dsg1b	-1.11	2.96E-01	↑Vim	**2.13**	5.87E-08
↓Dsg1c	-1.07	4.27E-01	↑Vtn	**1.65**	5.27E-06
↓Pkp1	1.02	5.46E-01	↓Bmp2	**-1.49**	1.02E-03
↑Pard6g	**1.55**	5.58E-04	↓Lrp6	**-1.14**	1.17E-03
↑Col1a1	**1.52**	2.47E-04	↓Tjp1	-1.15	9.09E-03
↑Col3a1	**1.87**	7.43E-05	↓Tjp2	-1.1	3.01E-02
↓Crb3	-1.1	7.71E-02	↓Tjp3	-1.1	2.61E-02
↑Ctnnb1	-1	6.14E-01	↓Tspan13	1.15	5.21E-02
↑Pard3	-1.07	1.85E-01	↑Timp1	**1.56**	1.51E-02
↑Pard6a	-1.12	1.68E-01	↑Cux1	-1.1	1.20E-02
↑Pard6b	-1.1	1.55E-01	↑Cd44	1.33	1.02E-02
↓Dsg2	-1.18	1.89E-03			
↓Dsg3	-1.07	2.38E-01			
↓Dsg4	-1.03	4.97E-01			
↓Dsp	-1.09	1.65E-02			
↓Epcam	-1.05	8.31E-02			
↓Flnb	-1.02	4.67E-01			
↑Fn1	**2.92**	1.42E-04			
↑Itga1	1.44	8.67E-03			
↑Itga2	1.09	3.01E-01			
↑Itga5	**1.69**	1.89E-02			
↓Itga6	1.03	4.16E-01			
↑Itgav	1.06	3.10E-01			
↓Jup	-1.03	2.79E-01			
↑Kat5	-1.04	2.93E-01			
↓Krt4	-1.06	4.41E-01			
↓Llgl2	-1.09	4.81E-02			
↑Loxl2	**1.77**	1.46E-04			
↑Mmp2	**2.68**	1.83E-07			
↑Mmp9	1.22	7.66E-02			
↓Mpp5	-1.17	3.58E-03			
↑Mst1r	-1.16	1.11E-02			
↓Muc1	**-1.53**	4.68E-02			
↑Ncam1	**1.52**	3.51E-04			
↑Ncam2	-1.03	5.65E-01			
↓Ocln	-1.21	1.05E-02			

References used: [1-6]

E.

Upstream Regulator	Molecule Type	Predicted Activation State	Activation z-score	p-value of overlap
TGFB1	growth factor	Activated	6.63	2.34E-53
Lipopolysaccharide	chemical drug	Activated	4.24	2.35E-38
Beta-estradiol	chemical - endogenous mammalian	Activated	2.88	1.07E-25
TP53	transcription regulator	Activated	2.11	2.83E-25
WNT3A	cytokine	Activated	2.07	7.85E-24

Table S3. (**A**) Table including the top 10 of significantly enriched up-regulated and (**B**) down-regulated gene sets between 12 and 28 months, as determined by GSEA. (**C**) Top 5 upstream regulators with the highest activation score between 12 and 28 months identified by IPA.

A.

NAME	SIZE	NES	FDR q-value
KEGG PHOSPHATIDYLINOSITOL SIGNALING SYSTEM	81	2.28	0.004
WP1259 RETINOL METABOLISM	39	2.21	0.005
SYNTHESIS OF PIPS AT THE PLASMA MEMBRANE	33	2.20	0.004
KEGG FATTY ACID DEGRADATION	46	2.14	0.007
KEGG INOSITOL PHOSPHATE METABOLISM	61	2.13	0.006
PI METABOLISM	50	2.09	0.009
WP401 MITOCHONDRIAL LC FATTY ACID BETA OXIDATION	16	2.07	0.010
BIOC GHPATHWAY	23	1.99	0.031
PHASE 1 FUNCTIONALIZATION OF COMPOUNDS	76	1.98	0.031
KEGG OTHER GLYCAN DEGRADATION	18	1.95	0.038

B.

NAME	SIZE	NES	FDR q-value
DNA REPLICATION	99	-2.77	<0.001
KEGG DNA REPLICATION	35	-2.75	<0.001
MITOTIC M/M G1 PHASES	280	-2.75	<0.001
SYNTHESIS OF DNA	92	-2.73	<0.001
SEPARATION OF SISTER CHROMATIDS	154	-2.72	<0.001
MITOTIC METAPHASE AND ANAPHASE	166	-2.71	<0.001
MITOTIC ANAPHASE	165	-2.7	<0.001
DNA REPLICATION PRE-INITIATION	77	-2.69	<0.001
M G1 TRANSITION	77	-2.69	<0.001
DNA STRAND ELONGATION	31	-2.67	<0.001

NES: Normalized Enrichment Score

C.

Upstream Regulator	Molecule Type	Predicted Activation State	Activation z-score	p-value of overlap
XBP1	transcription regulator	Inhibited	-5.34	2.65E-11
TBX2	transcription regulator	Inhibited	-3.31	4.15E-07
ERBB2	kinase	Inhibited	-3.42	1.33E-06
ATF6	transcription regulator	Inhibited	-3.13	1.45E-06
FBXO32	enzyme	Inhibited	-3.02	8.62E-06

Table S4. RNA integrity numbers (RIN) of the RNA isolated from colonic scrapings that were used for microarrays.

Mouse ID	Age (months)	RNA integrity number (RIN)
A1_03	6	10
A1_07	6	10
A1_09	6	9.9
A1_11	6	10
A2_01	12	9.9
A2_03	12	10
A2_05	12	9.9
A2_07	12	10
A2_09	12	9.9
A2_11	12	10
A2_13	12	10
A3_01	24	10
A3_03	24	10
A3_05	24	10
A3_07	24	10
A3_09	24	10
A3_11	24	10
A3_13	24	10
A3_15	24	10
A4_01	28	9.4
A4_03	28	10
A4_05	28	9.6
A4_09	28	9.6
A4_17	28	10
A4_19	28	9.8
A4_21	28	10
